# Small Molecule Oligopeptides Isolated from Walnut (*Juglans regia* L.) and Their Anti-Fatigue Effects in Mice

**DOI:** 10.3390/molecules24010045

**Published:** 2018-12-22

**Authors:** Rui Liu, Lan Wu, Qian Du, Jin-Wei Ren, Qi-He Chen, Di Li, Rui-Xue Mao, Xin-Ran Liu, Yong Li

**Affiliations:** 1 Department of Nutrition and Food Hygiene, School of Public Health, Peking University, Beijing 100191, China; lrui_pku@163.com (R.L.); wulan_5@163.com (L.W.); duqian_an_1991@163.com (Q.D.); ren_en_jinwei@126.com (J.-W.R.); qiheyuntian@163.com (Q.-H.C.); lidiyy@126.com (D.L.); maoruixue@163.com (R.-X.M.); liuhappy07@163.com (X.-R.L.); 2 Medical Center, SinoMed Peptide Valley Bioengineering Co., Ltd., Beijing 100027, China

**Keywords:** walnut oligopeptides, anti-fatigue, weight-loaded swimming

## Abstract

Walnut (*Juglans regia* L.) is unique for its extensive biological activities and pharmaceutical properties. There are few studies on walnut oligopeptides (WOPs), which are small molecule peptides extracted from walnuts. This study aimed to evaluate the anti-fatigue effects of WOPs on ICR mice and explore the possible underlying mechanism. Mice were randomly divided into four experimental sets and each set of mice were then randomly divided into four groups. The vehicle group was administered distilled water, and the three WOP intervention groups were orally administered WOP solution at a dose of 110, 220, and 440 mg/kg of body weight, respectively. After 30 days of WOP intervention, the anti-fatigue activity of WOPs were evaluated using the weight-loaded swimming test and by measuring the change of biochemical parameters, glycogen storage and energy metabolism enzymes, anti-oxidative capacity and mitochondrial function. It was observed that WOPs could significantly prolong the swimming time, decrease the accumulation of lactate dehydrogenase (LDH), creatine kinase (CK), blood urea nitrogen (BUN) and blood lactic acid (BLA), and increased the glycogen storage of liver and gastrocnemius muscle. WOPs also markedly inhibited fatigue induced oxidative stress by increasing the activity of superoxide dismutase (SOD), glutathione peroxidase (GPX) and decreasing the content malondialdehyde (MDA). Notably, WOPs improved the activity of pyruvate kinase (PK), succinate dehydrogenase (SDH), Na+-K+-ATPase, and enhanced the mRNA expression of mitochondrial biogenesis factors and mitochondrial DNA content in skeletal muscles of mice. These results suggest that WOPs have beneficial anti-fatigue effects, which may be attributed to their positive effects on increasing glycogen storage, improving energy metabolism, inhibiting oxidative stress, enhancing mitochondrial function in skeletal muscle, and ameliorating the cell damage and the muscular injury.

## 1. Introduction

Fatigue is a common non-specific symptom defined as an overwhelming sense of tiredness, weakness or exhaustion, which can cause a broad range of physical and mental discomfort. There are various kinds of diseases related to fatigue, such as cancer, multiple sclerosis, hypertension, depression, diabetes, coronary heart disease, and Parkinson’s disease, as well as inflammation [1,2,3,4]. Long-term accumulated fatigue can induce overwork, chronic fatigue syndrome, and even karoshi (unexplained sudden death associated with overwork) [5,6]. In modern society, with the increasingly fierce social competition that accelerate the pace of life and a prevalence rate of 0.4%–1, more than 70 million people worldwide suffering from fatigue and fatigue-related adverse effects of health. Fatigue affects over 800,000 people in America and a population prevalence of at least 0.2% has been reported in the UK [3,4,6,7,8]. However, most of these people are not receiving effective therapy to relieve fatigue; in part because the pathophysiological mechanisms and etiology of fatigue remain unclear [3,7]. Thus, finding effective and safe anti-fatigue methods are necessary and significant.

In the past few decades, physicians and researchers have been looking for natural active substances and synthetic chemicals to enhance physical fitness, postpone fatigue, and accelerate the elimination of fatigue and recovery from physical exertion in people, especially for athletes [3,9]. However, many of the active products are limited due to side effects. In recent years, nutritional intervention and treatment gradually attracted researchers’ attention, various studies showed that active ingredients extracted from natural foods are effective and safe in preventing and alleviating fatigue.

For instance, small-molecule oligopeptides isolated from *Panax quinquefolium* L. can inhibit oxidative stress and improve mitochondrial function in skeletal muscles [10], Sake protein supplementation can enhance athletic performance and resistance to fatigue [11], and polysaccharide-rich extract from corn silk can relieve fatigue with high safety [12].

Walnut (*Juglans regia* L.) is one of the mopst widespread tree nuts in the world. It is rich in fat content as well as proteins, vitamins and minerals [13]. Walnut has extensive health-promoting functions, including antifungal [14], antitumor [14], antioxidant [13], anti-inflammatory [15], immune modulation [14], and antihypertensive properties [16]. Bioactive peptides are important protein fragments that have a wide range of pharmacological functions, such as anti-oxidative, anti-hypoxia, antimicrobial and immunomodulatory [17]. One animal study showed that oral administration of walnut extract had anti-fatigue effects and could significantly elevate exercise tolerance in mice [18]. However, research is lacking on walnut oligopeptides (WOPs), which are the general name for small molecule oligopeptides extracted from walnut, with low molecular weight, absorbable features and high bioavailability, that have plenty of potential physiological activities [8,19]. Little is currently known about the anti-fatigue effects of WOPs. Thus, further study about the anti-fatigue function of small molecule oligopeptides isolated from walnut (WOPs) is warranted. In the present study, after ICR mice were treated with WOPs for 30 days, weight-loaded forced swimming time, biochemical parameters, antioxidant enzymes, energy metabolic factors, and mitochondrial activity were measured to evaluate the anti-fatigue effects of WOPs and elucidate the possible underlying mechanisms.

## 2. Results

### 2.1. Effects of WOPs on the Body Weight of Mice

The effects of WOPs on the body weight are presented in Table 1. There were no statistical significance in body weight changes among the vehicle group and three WOPs intervention groups in the Experimental Sets 1, 2, 3 and 4, respectively.

### 2.2. Effects of WOPs on Weight-Loaded Forced Swimming Endurance Time

**Figure 1:**
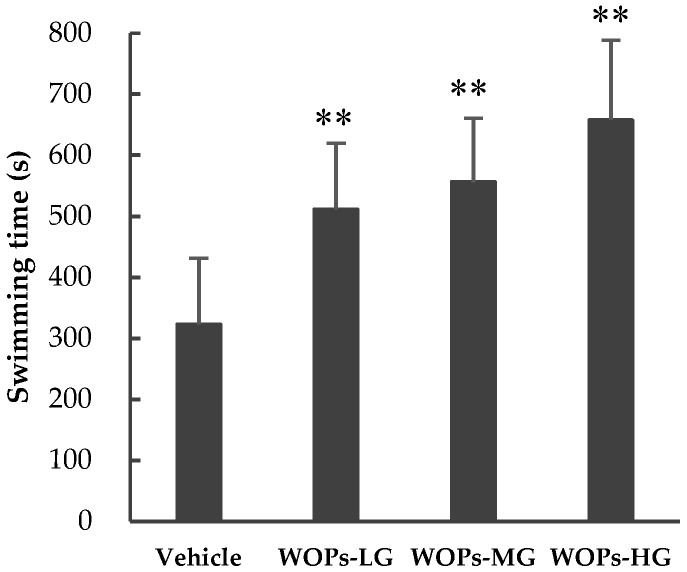
Effects of WOPs on weight-loaded forced swimming endurance time in mice. All the values are presented as mean ± SD (*n* = 10), ** *p* < 0.01 versus vehicle group. WOPs-LG, WOPs-MG, WOPs-HG refer to that mice were treated with walnut oligopeptides (WOPs) at 110, 220 and 440 mg/kg for 30 days, respectively.

The weight-loaded forced swimming test is the most commonly used animal model to evaluate the extent of fatigue [3]. A longer swimming time indicates the increase of exercise capacity and reduced susceptibility to fatigue [20,21]. As shown in Figure 1, in comparison with the vehicle group (323.40 ± 107.99), the swimming time of mice in in all three WOPs groups was longer (WOPs-LG: 511.65 ± 107.58; WOPs-MG: 556.33 ± 104.13; WOPs-HG: 657.53 ± 130.90) and the difference was statistically significant (*p* < 0.01). These results suggested that WOPs had significant anti-fatigue activity and could enhance exercise performance.

### 2.3. Effects of WOPs on Lactate Dehydrogenase (LDH), Blood Urea Nitrogen (BUN), Creatine Kinase (CK) and Blood Glucose Content

**Figure 2:**
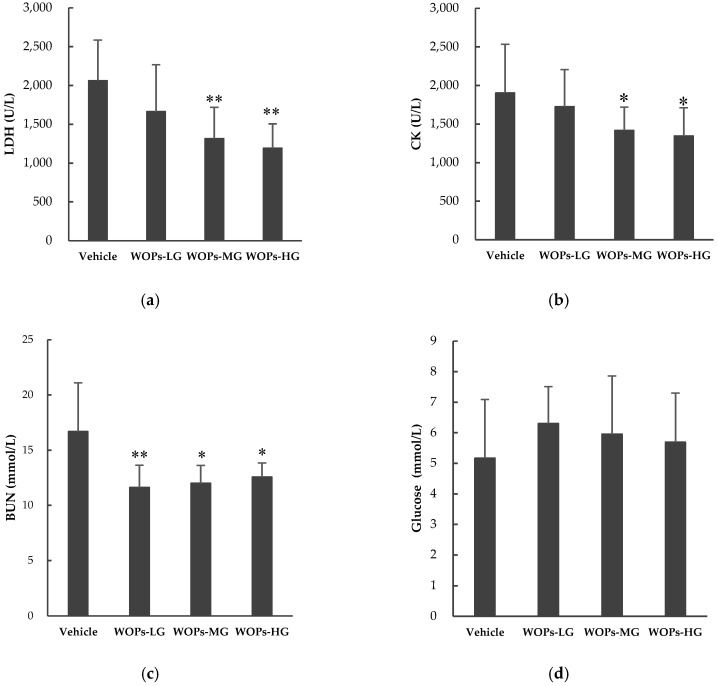
The effects of WOPs on serum biochemical parameters including (**a**) lactate dehydrogenase (LDH); (**b**) creatine kinase (CK); (**c**) blood urea nitrogen(BUN); and (**d**) blood glcouse. Data are expressed as mean ± SD (*n* = 10). * *p* < 0.05, ** *p* < 0.01 indicate significant difference versus vehicle group. WOPs-LG, walnut oligopeptides low-dose group at a dose of 110 mg/kg; WOPs-MG, walnut oligopeptides medium-dose group at a dose of 220 mg/kg; WOPs-HG, walnut oligopeptides high-dose group at a dose of 440 mg/kg.

Several blood biochemical indicators were used to evaluate the degree of muscle fatigue and to explore the underlying mechanism, such as lactate dehydrogenase (LDH), blood urea nitrogen (BUN), creatine kinase (CK) and blood glucose [3,20]. In the present study, we examined whether WOPs could cause positive effects on these related biochemical parameters in the serum sample of mice (Figure 2). After 30 days of treatment, compared with the vehicle group, the activities of LDH, CK were significantly decreased in WOPs-MG and WOPs-HG (*p* < 0.01 for LDH activity, *p* < 0.05 for CK activity) (Figure 2a,b), and the BUN levels were markedly decreased in all three WOPs groups (*p* < 0.01 for WOPs-LG, *p* < 0.05 for WOPs-MG and WOPs-HG) (Figure 2c). There were no significant difference in the contents of blood glucose between the vehicle group and all WOPs group (*p* > 0.05) (Figure 2d).

### 2.4. Effects of WOPs on Blood Lactic Acid (BLA) Levels in Mice

For the high correlation between muscle and blood lactate, the accumulation of blood lactic acid (BLA) is an important indicator to evaluate the speed and extent of fatigue development [22,23,24]. The effects of WOPs in BLA concentrations at different time points are presented in Table 2. There were no obvious difference of BLA concentrations at baseline among the groups (*p* > 0.05). The concentrations of BLA in WOPs-HG were significantly lower than that of the vehicle group at 0 min after swimming (*p* < 0.05). At 20 min after swimming, the BLA values from three WOPs groups were significantly decreased compared to the vehicle group (*p* < 0.01). In addition, the area under BLA curve was remarkably reduced in WOPs-LG and WOPs-HG in comparison with the vehicle group (*p* < 0.01).

### 2.5. Effects of WOPs on Hepatic and Muscular Glycogen Level

**Figure 3:**
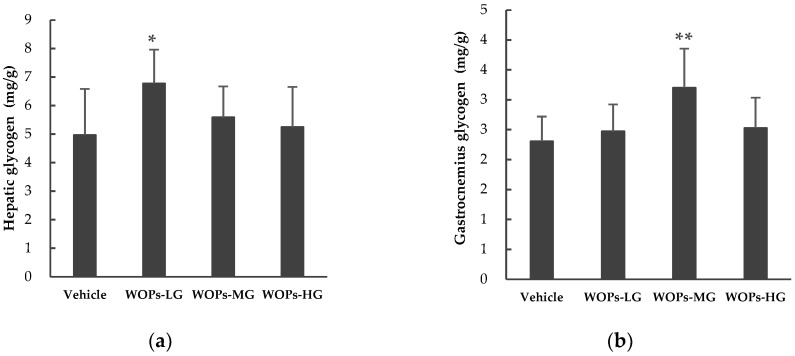
Effects of WOPs on liver glycogen (**a**) and gastrocnemius glycogen (**b**) in mice. Data are mean ± SD (*n* = 10). * *p* < 0.05, ** *p* < 0.01 indicate significant difference compared with the vehicle group. WOPs-LG, 110 mg/kg of walnut oligopeptides group; WOPs-MG, 220 mg/kg of walnut oligopeptides group; WOPs-HG, 440 mg/kg of walnut oligopeptides group.

The glycogen storage is of great importance in enhancing exercise capacity and duration, and directly reflecting the level of fatigue [22,25,26]. After the mice administrated with WOPs for 30 days, the glycogen content of the liver and gastrocnemius were examined. As shown in Figure 3, the contents of hepatic glycogen in WOPs-MG and WOPs-HG (Figure 3a), and gastrocnemius glycogen levels in WOPs-LG and WOPs-HG (Figure 3b) showed minimal increased (*p* > 0.05). Moreover, there were a significant difference between the hepatic glycogen contents in WOPs-LG and the vehicle group (*p* < 0.05), and the gastrocnemius glycogen levels were markedly increased in WOPs-MG (*p* < 0.01).

### 2.6. Effects of WOPs on PK, SDH, and Na+-K+-ATPase Activities in Skeletal Muscles of Mice

**Figure 4:**
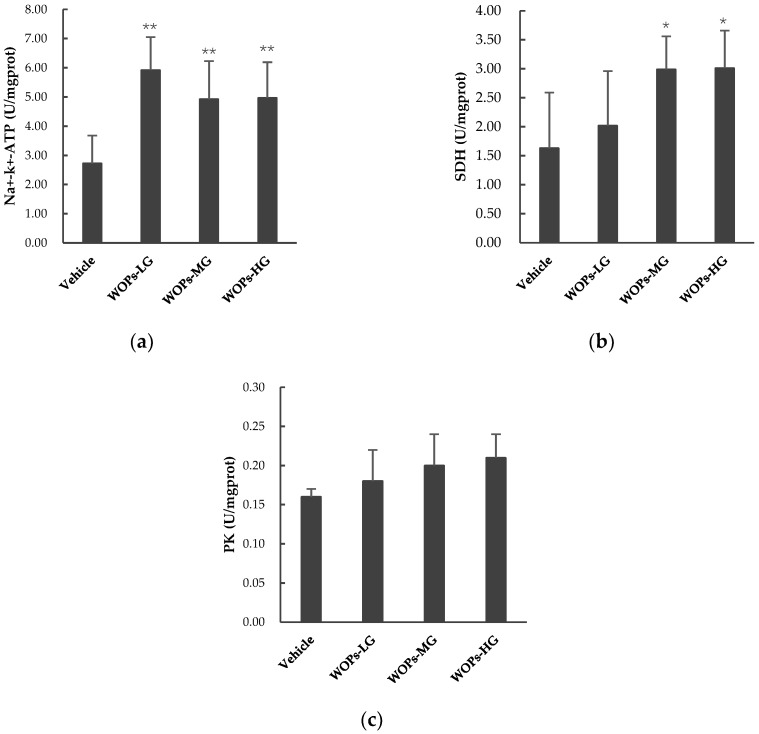
The regulation effects of WOPs on the activity PK, SDH, and Na+-K+-ATPase in the skeletal muscle of mice. (**a**) Na+-K+-ATPase; (**b**) pyruvate kinase (PK); (**c**) succinate dehydrogenase (SDH). Values are expressed as mean ± SD (*n* = 10). * *p* < 0.05, ** *p* < 0.01 mean significant difference versus vehicle group. WOPs-LG, walnut oligopeptides low-dose group at a dose of 110 mg/kg; WOPs-MG, walnut oligopeptides medium-dose group at a dose of 220 mg/kg; WOPs-HG, walnut oligopeptides high-dose group at a dose of 440 mg/kg.

To further explore the effects of WOPs on the main processes of energy metabolism, we examined the activities of key energy metabolic enzymes, including pyruvate kinase (PK), succinate dehydrogenase (SDH), and Na+-K+-ATPase in skeletal muscles of mice. WOPs significantly increased the activity of Na+-K+-ATPase (Figure 4a) and SDH (Figure 4b) in muscles of mice compared with vehicle group (*p* < 0.01, *p* < 0.05), whereas there were no significant differences in PK among the four groups (*p* > 0.05) (Figure 4c). These results suggest that WOPs can effectively regulate energy metabolism and antagonize fatigue.

### 2.7. Effects of WOPs on Parameters of Oxidative Stress in Skeletal Muscles of Mice

Ample evidence suggests that intense and prolonged exercise can produce high levels of reactive oxygen species, inducing free-radical-mediated oxidative damage to tissues and resulting in muscle weakness and fatigue [27]. Biomarkers to evaluate the antioxidant capacity and oxidative damage in muscle fatigue include superoxide dismutase (SOD), glutathione peroxidase (GPX) and malondialdehyde (MDA), etc. [5].

As shown in Table 3, in comparison with the vehicle group, the activities of SOD and GPX were improved significantly in WOPs-MG and WOPs-HG (*p* < 0.01); in addition, the MDA levels were remarkably attenuated in WOPs-MG and WOPs-HG (*p* < 0.01).

### 2.8. Effects of WOPs on Mitochondrial Biogenesis Factors and mtDNA Copy Number in Skeletal Muscles

**Figure 5:**
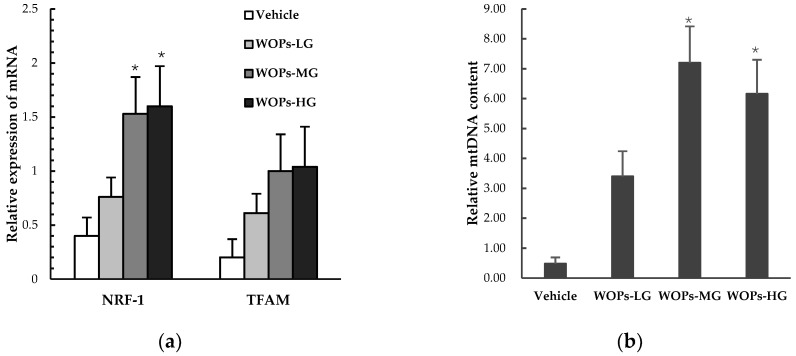
Effects of WOPs on the RNA expression of NRF-1, TFAM (**a**) and mtDNA copy number (**b**) in skeletal muscles of mice by real-time PCR analysis. β-actin mRNA levels were used as a control. Data are reprensated as means ± SD (*n* = 10). * *p* < 0.05 versus vehicle group. NRF-1, Nuclear respiratory factor 1; TFAM, Mitochondrial transcription factor A; mtDNA, mitochondrial DNA. WOPs-LG, 110 mg/kg of walnut oligopeptides group; WOPs-MG, 220 mg/kg of walnut oligopeptides group; WOPs-HG, 440 mg/kg of walnut oligopeptides group.

Both the nuclear respiratory factor 1(NRF-1) and mitochondrial transcription factor A(TFAM) were mitochondrial biogenesis factors considered essential for mitochondrial gene expression in mammals, the result showed that the mRNA expression of NRF-1was markedly increased in WOPs-MG and WOPs-HG groups compared with the vehicle group (*p* < 0.05) (Figure 5a). In addition, the mitochondrial DNA (mtDNA) copy number was also significantly improved in WOPs-MG and WOPs-HG groups after the WOPs treatment (*p* < 0.05) (Figure 5b).

## 3. Discussion

Walnut (*Juglans regia* L.) consumption has been widely recommended for a long history due to its abundant bioavailability, and varied biological activity, including antifungal, anti-inflammatory and hypotensive properties and antioxidant activities [13,14,18,28]. Much attention has been focused on walnut oil, while studies investigating the biological activities of WOPs, which include small molecule oligopeptides isolated from walnut, are however rare. The present study demonstrated that oral supplementation of WOPs could enhance swimming endurance capacity and storage of liver and muscle glycogen, reduce the levels of LDH, CK, BUN and BLA, and simultaneously inhibit fatigue induced oxidative stress in mice. In addition, the anti-fatigue effect of WOPs may be associated with the enhancement of energy metabolism and the improvement of mitochondrial activity.

Repeated, intense physical labor results in a decline in performance known as fatigue, which can induce a series of pathological and physiological impairments and alterations, and causing mechanism dysfunction including endocrine, immune, and metabolic imbalance [29,30]. The weight-loaded forced swimming test is a valid experimental protocol used widely for reflecting the degree of exercise of endurance and fatigue in laboratory animals, and screening the anti-fatigue activities of certain agents [4,20,31,32]. In the study, the results of weight-loaded swimming test demonstrated that WOPs treatment significantly prolonged the time to exhaustion of mice, indicating that WOPs possesses an anti-fatigue effect, and 440 mg/kg is the optimal dose, which showed the longest mean swimming time when compared with the vehicle group (Figure 1).

To further reflect the real status of fatigue in mice and evaluate the anti-fatigue properties of WOPs, several biochemical indexes derived from blood and tissues that contribute to the fatigue were analyzed. LDH is involved in anaerobic glycolysis and gluconeogenesis by promoting the redox reaction between lactic acid and pyruvic acid, and its reaction is the basis of the lactic acidosis construct [33,34]. CK is a critical kinase directly relates to the intracellular energy transfer, muscle contraction and ATP regeneration, which can be used in the diagnosis of muscular dystrophy and myocardial infraction [35]. However, during the exhaustion exercise, excessive production of free radicals can lead to myocyte damage and leakage of LDH and CK into serum [33]. Therefore, as cytosolic enzymes, both the elevation of LDH and CK can serve as critical indexes for reflecting the cell damage and the subsequent extent of muscle disruption induced by intense exercise [3,20,33,36]. BUN is the main product of protein and amino acid metabolism [31]. Along with the increasing intense of exercise, the energy derive from carbohydrates and fats is not sufficient for the body, and then protein and amino acids become alternative sources for catabolic metabolism to compensate the energy requirements, which causes an increase in BUN [8,31,33]. Thus, increased BUN levels in serum commonly represents the impairment of the contractive strength of muscle and typical indictors related to fatigue [33]. Our result indicated that WOPs had a positive effect to decrease that accumulation of LDH, CK and BUN (Figure 2). The results suggested that the supplementation of WOPs could mitigate the cell damage and the muscular injury induced by fatigue.

During high-intensity exercise, when the oxidative phosphorylation fails to meet the energy needs, the aerobic metabolism transfer to anaerobic glycolysis or glycogenolysis, thereby causing the accumulation of lactate [5,37,38]. The accumulated lactate in the muscles or blood further resulting in a decrease in cellular pH, which could induce muscle fatigue and affects the feeling of pain and discomfort [34,37,39]. There was a remarkable positive correlation between the blood lactate clearance rate and alleviate muscle damage and recovery from fatigue [25,36]. In addition, glycogen is the principle store of carbohydrate energy for ATP generation, which primarily exists in muscle and liver cells [40]. There is now compelling evidence that glycogen is the main substrate to maintain energy transduction during aerobic exercise and oxidative phosphorylation [40,41]. This may also explain the importance of glycogen as a fuel that can improve exercise endurance and delay fatigue [29,40]. In the present study, the results showed that compared with the vehicle group, the concentration of BLA dramatically reduced (Table 2) and the glycogen storage in the liver and skeletal muscles improved (Figure 3) in the WOPs administrated groups. These findings are consistent with the previous studies, which reported similar results in walnut extracts [18]. Furthermore, fatigue and energy are inextricably associated, in order to further verify the effect of WOPs on improving the energy metabolism in the skeletal muscle, several energy metabolic enzymes include pyruvate kinase (PK), succinate dehydrogenase (SDH), and Na+-K+-ATPase were examined [10,20,42]. The Na+-K+-ATPase in skeletal muscle is rapidly stimulated during strenuous exercise, which plays a vital role in maintenance of muscle contraction and fatigue resistance [43]. PK and SDH are two rate-limiting enzymes involved in the regulation of glycolysis pathway and tricarboxylic acid cycle, which are absolutely required to catalyze ATP synthesis [42]. In the study, the results showed that WOPs could also enhance the activity of Na+-K+-ATPase, and SDH in the skeletal muscle of mice with fatigue. Thus, these findings on energy metabolic enzymes clearly illustrate an effective role of WOPs in the regulation of energy metabolism inhibited by the oxidative stress related to fatigue.

Physical exercise leads to free radical-mediated damage to tissues as first reported in 1978. It is now well established that strenuous exercise induces redox disturbances in skeletal muscle that can reduce force production and accelerate fatigue [27,44]. Importantly, growing evidence suggests that overproduced free radicals can damage proteins, lipids and DNA in the contracting myocytes, mediate activation of calpains and caspases, and modulate cellular signaling pathways including the mitogenactivated protein kinase (MAP kinase) and nuclear factor κ-B (NFκB) pathways [27,32]. The superoxide dismutase (SOD) and glutathione peroxidase (GPX) are the principal endogenous antioxidant enzymes to attenuate the damage induced by excessive oxidative stress, which by scavenging free radicals and their metabolites [8,27,45]. Malondialdehyde (MDA) is one of the degradation products of membrane lipid peroxidation caused by free radicals, which is an important indicator for evaluating cellular oxidative stress [8,33]. Consequently, adequate SOD and GPX activities, low level of MDA are regarded as essential for preventing and reducing oxidative damage induced by fatigue [33]. In this study, WOPs treatment significantly elevated SOD and GPX activities while decreasing MDA contents (Table 3). Thereby suppressing oxidative stress and generating more ATP for energy supplement might be a potential mechanism of the effects of WOPs against fatigue.

Mitochondria is one of the most important organelles which involved in a plethora of fundamental life processes, including energy conversion, Kreb’s cycle, oxidative phosphorylation, and modulation of calcium homoeostasis, etc. [46]. Therefore, mitochondrial dysfunction (EIMD) can affect a variety of tissues and preferentially affect muscles and nerves, which is responsible for multiple adverse consequences including cardiac dysfunction, chronic fatigue and overtraining syndrome [46,47]. The role of physical activity in improving mitochondrial function in skeletal muscle was well established in the previous studies [46,48,49]. However, recent studies in both human and rodents have documented that excessive exercise may lead to EIMD in skeletal muscle of human and experimental animals, and the similar detrimental effect on mitochondrial function was also found in the brain, heart, liver and blood cells [46]. Thus, the nuclear respiratory factor 1 (NRF-1), mitochondrial transcription factor A (TFAM), and mitochondrial DNA (mtDNA) copy number in the skeletal muscle of mice were measured to evaluated the effects of WOPs on mitochondrial function. As a positive transcriptional regulator, NRF-1 plays a key role in the regulation of mitochondrial biosynthesis and other biological functions, including signal transduction, organelle biogenesis, protein synthesis, and cell cycle progression [8,50]. Among the specific effects of relevance, TFAM promotes transcription and replication of mitochondrial DNA and is up-regulated by NRF-1 [50]. MtDNA copy number was considered as a functional marker of mitochondrial function, and hard to recover its deletions [8,10]. In the present study, we found WOPs could improve the mRNA expression of NRF-1 and restoring the mtDNA content in skeletal muscles of mice. These findings strongly indicate that the anti-fatigue effect of WOPS may possibly be due to its positive effects on improving mitochondrial function during intense exercise. Moreover, human and animal studies have shown that serum branched-chain amino acids (BCAA) levels, including leucine, isoleucine, and valine, were decreased after exhaustive exercise, and BCAA and arginine supplementation could improve performance in exercise by potentially alleviating central fatigue [6,51,52]. Our results show that the content of free amino acids in WOPs was 2.98%, the amino acids composition analysis demonstrated WOPs samples to be rich in arginine (Arg) > phenylalanine (Phe) > leucine (Leu) > alanine (Ala) > glutamic acid (Glu) (Table 4). Therefore, it may also be one of the mechanisms that WOPs could alleviate and against fatigue.

## 4. Materials and Methods

### 4.1. Preparation and Identification of WOPs

The WOPs sample, which was derived from the residual protein of Walnut (*Juglans regia* L.) by enzymatic hydrolysis, was provided by Tianpeptide Biotechnology Co. Ltd. (Tianjin, China). In brief, after initial cleansing, mincing, and homogenization in distilled water, it was added to complex protease (3000 U/g protein) at 40 °C for 3 h after adjusting the pH to 8.0 using sodium hydroxide. Then, WOP powders were obtained mainly by activated carbon adsorption, nanofiltration, cryoconcentration, decolorization, purification and spray drying.

The oligopeptides sample was purified by High-Performance Liquid Chromatography (HPLC, Water Corp., Milford, MA, USA) using a Phenomenex C18 column (10 mm × 250 mm), and the molecular weight distribution of the WOPs sample was measured by LDI-1700 matrix-assisted laser desorption ionization time-of-flight mass spectrometry (MALDI-TOF-MS, Liner Scientific Inc., Reno, NV, USA). Then, we measured the molecular weight distribution along with amino acid composition analysis using the automatic amino acid analyzer (Hitachi, Tokyo, Japan). Using the HPLC to estimate the amount of free amino acids, the small molecule oligopeptides (defined as relative molecular weight was between 180 and 1000 in WOPs was 86.5%, and free amino acids comprised 2.98%. The amino acids composition analysis demonstrated WOPs samples to be rich in arginine (Arg) > phenylalanine (Phe) > leucine (Leu) > alanine (Ala) > glutamic acid (Glu). The amino acid composition is shown in Table 4.

### 4.2. Chemicals and Reagents

Assay kits used for the determination of blood urea nitrogen (BUN), lactate dehydrogenase (LDH), creatine kinase (CK), and blood glucose were purchased from Yingkexinchuang Science and Technology Ltd. (Macau, China). The detection kits of blood lactic acid (BLA), liver/muscle glycogen, superoxide dismutase (SOD), glutathione peroxidase (GPX), malondialdehyde (MDA), pyruvate kinase (PK), succinate dehydrogenase (SDH), and Na+-K+-ATPase were purchased from Nanjing Jiancheng Biotechnology Institute (Nanjing, China). All other reagents used in this study were of analytical grade.

### 4.3. Animals and Experimental Design

One hundred and sixty male Institute of Cancer Research (ICR) mice (6–8 weeks old, 18–22 g) in a specific pathogen-free condition were obtained from Animal Service of Health Science Center, at Peking University. The mice were housed at a constant temperature (25 ± 1 °C) and humidity (50–60%) under a 12 h: 12 h light-dark cycle with free access to standard food (American Institute of Nutrition Rodent Diets-93G (AIN-93G diet) and water. The experiment was reviewed and approved by the Institutional Animal Care and Use Committee of Peking University, and all animals were treated according to the Principles of Laboratory Animal Care (NIH publication No. 85-23, revised 1985) and the guidelines of Peking University Animal Research Committee (Laboratory animal production license No.: SCXK (Jing) 2016-0010; Laboratory animal use license No.: SYXK (Jing) 2016-0041.

After one week of acclimatization, the mice were randomly divided into four experimental sets (*n* = 40). Each sets of mice were further divided into four intervention groups (*n* = 10): (1) vehicle group (distilled water); (2) 110 mg/kg WOPs (WOPs-LG); (3) 220 mg/kg WOPs (WOPs-MG); and (4) 440 mg/kg WOPs (WOPs-HG). The mice in WOPs groups were administrated orally via the WOPs solution (dissolve the difference dose of WOPs in distilled water), while distilled water was administered orally to mice in vehicle control group. Changes in the body weight of mice were recorded once a week. The animals were administrated once a day for 30 consecutive days, and then were used for further experiments.

### 4.4. Weight-Loaded Swimming Test

Mice from Experimental Set 1 were used for the weight-loaded forced swimming test. The test was carried out followed previous studies described by Bao and Tan [8,53]. Briefly, mice in Set 1 were placed individually in the swimming pool (50 × 50 × 40 cm) filled with water (25 ± 1 °C) to a depth of 30 cm so that mice could not touch the bottom of the swimming pool with their feet to support themselves. A lead sheath (5% of body weight) was attached to the tail root of the mouse. The swimming time was recorded immediately when the physical strength of the mouse was assessed to be exhausted and it failed to rise to the surface of water for more than 10 s.

### 4.5. Biochemical Assay

The analysis of biochemical parameters were determined in mice from Experimental Set 2. Typically, mice in Set 2 were forced to swim in water at 30 °C without any loads for 90 min, then they were placed back to their home cages. After resting for one hour, blood was obtained by removing the eyeball, and then heart, liver and skeletal muscles (quadriceps femoris of both hind legs) were immediately dissected and frozen at −80 °C. Serum was prepared by centrifugation at 3000 rpm at 4 °C for 15 min. The content of lactate dehydrogenase (LDH), blood urea nitrogen (BUN), creatine kinase (CK) and glucose in the serum were measured by an automatic biochemical analyzer (Olympus Corporation, Tokyo, Japan). The activities of superoxide dismutase (SOD), glutathione peroxidase (GPX), pyruvate kinase (PK), succinate dehydrogenase (SDH), malate dehydrogenase (MDH), and Na+-K+-ATPase in skeletal muscles were determined by detection kits as listed in Section 4.2.

### 4.6. Determination of Blood Lactic Acid

The concentrations of BLA were measured in mice belonging to the Experimental Set 3. The mice in set 3 were forced to swim in water for 10 min under the condition of no load at 30 °C. Accurate blood samples was collected at three time points: at baseline, 0 min after swimming, and 20 min after swimming. Twenty µL of blood was precisely extract from the angular vein of mice by a glass capillary each time, and then immediately poured into the bottom of a 5 mL centrifuge tube already containing 0.48 mL of 1% sodium fluoride solution. The glass capillary was flushed several time with the supernatants. Then the levels of BLA were determined according to the procedures specified in the kits. The area under the BLA curve was calculated according to the following Equation (1):

C_S_ = 1/2 × (C_0_ + C_1_) × 10 + 1/2 × (C_1_ + C_2_) × 20 (1)

where C_0_, C_1_, and C_2_ stand for the BLA concentration of mice at baseline, 0 and 20 min after swimming, respectively. Cs stands for the area under the BLA curve.

### 4.7. Examination of Glycogen in Liver and Skeletal Muscles

Mice from Experimental Set 4 were used to examine the content of glycogen in liver and skeletal muscles. Thirty days after the intervention of WOPs, the mice in Set 4 were sacrificed, their livers and skeletal muscles (quadriceps femoris of both hind legs) were immediately isolated and homogenized to 10% solution with normal saline at 4 °C. Then the tissue glycogen contents were measured following the recommended procedures provided by the detection kits.

### 4.8. Quantitative Real-Time PCR and Analyses of mtDNA Content

Total RNA and DNA were extracted from isolated skeletal muscles of mice from Experimental Set 2 by Trizol reagent (Invitrogen, Carlsbad, CA, USA) and DNeasy Tissue Kit (QIAGEN Sciences, Germantown, MD, USA), respectively. Real-time reverse transcription-PCR was performed using ABI PRISM 7500 real-time PCR detection system to detect the RNA expression of target genes with the specie primers, nuclear respiratory factor 1 (NRF-1), Forward 5′-TATGGCGGAAGTAATGAAA GACG-3′ and Reverse 5′-CAACGTAAGCTCTGCCTTGTT-3′; mitochondrial transcription factor A (TFAM), Forward 5′- AGGTCCAGCTCACTAACTGC -3′ and Reverse 5′-TGTATGCTGTGGTT TCCCAGT-3′; β-actin, Forward 5′-GATTACTGCTCTGGCTCCTAG -3′ and Reverse 5′- GACTCAT CGTACTCCTGCTTGC -3′. Real-time PCR was used to detect mtDNA copy number. The sequences are as follows: Mitochondrial DNA (mtDNA), Forward 5′- CGTTAGGTCAAGGTGTAGCC -3′ and Reverse 5′-CCAGACACACTTTCCAGTATG-3′; β-actin, Forward 5′-GATTACTGCTCTGGCTCC TAG -3′ and Reverse 5′- GACTCATCGTACTCCTGCTTGC -3′. Cycling conditions were pre-denaturation at 95 °C for 2 min followed by 35 repeats of denaturation at 95 °C for 15 s and annealing for 30 s. Target mRNA values and the content of mtDNA copy number were determined by comparison to the control sample after being normalized to β-actin levels and calculated using the comparative cycle threshold (^△△^Ct) method.

### 4.9. Statistical Analysis

Statistical analyses were performed using the SPSS software version 24 (SPSS Inc., Chicago, IL, USA). All values were presented as mean ± standard deviation (SD). Differences between groups were analyzed by one-way analysis of variance test and LSD methods if the data were homogeneous or Tamhane’s T3 test if variances were unequal. A value of *p* < 0.05 was considered statistically significant.

## 5. Conclusions

In this study, WOPs, small molecule oligopeptides extracted from walnut (*Juglans regia* L.), have been used to study their anti-fatigue effect on ICR mice. The results demonstrated that WOPs could prolong the weight-loaded forced swimming time, delay the accumulation of LDH, CK, BUN and BLA, increase glycogen storage in liver and skeletal muscle and improve energy metabolism enzymes includes Na+-K+-ATPase and SDH. Moreover, WOPs could enhance anti-oxidation capacity and mitochondrial function in skeletal muscles of mice, and ameliorate the cell damage and the muscular injury, thereby against fatigue. These findings suggested that WOPs could be a novel natural agent for prevent and against fatigue. Ongoing research continues to probe the exact molecular mechanism by which WOPs against fatigue and explore the biological effectiveness and optimal dose of WOPs to generate their anti-fatigue effects in humans.

## Figures and Tables

**Table 1:** Effects of WOPs on the body weight in mice.

Body Weight (g)	Vehicle	WOPs-LG	WOPs-MG	WOPs-HG
**Set 1**				
Initial body weight	24.62 ± 2.04	24.88 ± 1.64	25.47 ± 1.98	24.73 ± 1.23
Final body weight	38.69 ± 3.26	38.75 ± 2.57	38.37 ± 3.19	39.65 ± 2.82
**Set 2**				
Initial bogy weight	25.50 ± 1.43	25.16 ± 1.42	24.98 ± 1.47	25.14 ± 1.48
Final body weight	39.01 ± 2.52	39.33 ± 1.41	39.94 ± 2.80	41.12 ± 3.13
**Set 3**				
Initial body weight	26.40 ± 1.42	25.27 ± 1.94	25.19 ± 1.77	24.73 ± 1.77
Final body weight	39.02 ± 3.00	39.23 ± 2.17	38.58 ± 2.71	39.07 ± 3.21
**Set 4**				
Initial body weight	25.88 ± 1.43	25.42 ± 1.56	25.08 ± 1.04	25.19 ± 1.57
Final body weight	39.03 ± 2.12	40.16 ± 2.82	39.40 ± 3.20	39.73 ± 3.27

Values are expressed as mean ± SD for *n* = 10 mice in each group. Walnut oligopeptides low-dose group (WOPs-LG), walnut oligopeptides medium-dose group (WOPs-MG) and walnut oligopeptides high-dose group (WOPs-HG) at 110, 220 and 440 mg/kg, respectively.

**Table 2:** Effects of WOPs on the level of BLA at different time points in mice.

BLA (g/L)	Vehicle	WOPs-LG	WOPs-MG	WOPs-HG
Baseline	6.04 ± 0.93	6.09 ± 0.79	6.37 ± 2.07	6.46 ± 1.77
0 min after swimming	7.65 ± 1.23	7.40 ± 0.59	8.49 ± 1.08	6.55 ± 1.56 *
20 min after swimming	7.99 ± 0.92	5.10 ± 0.44 **	4.83 ± 0.37 **	4.63 ± 0.63 **
Area under BLA curve	224.89 ± 21.48	192.43 ± 7.91 **	206.89 ± 19.98	173.66 ± 14.99 **

Values are expressed as mean ± SD, *n* = 10 for each group. BLA, blood lactate. * *p* < 0.05, ** *p* < 0.01 indicate significant difference versus vehicle group. WOPs-LG, WOPs-MG, WOPs-HG refers to three walnut oligopeptides groups with dose of 110, 220 and 440 mg/kg, respectively.

**Table 3:** Effects of WOPs on SOD, GSH-PX activity, and MDA contents in skeletal muscles of mice.

Groups	SOD (U/L)	GPX (U/L)	MDA (nmol/L)
Vehicle group	129.97 ± 12.78	564.79 ± 48.33	3.42 ± 0.32
WOPs-LG	139.29 ± 13.95	530.85 ± 65.48	3.25 ± 0.28
WOPs-MG	177.23 ± 19.14 **	661.47 ± 45.13 **	2.11 ± 0.39 **
WOPs-HG	185.75 ± 15.13 **	685.86 ± 57.63 **	2.34 ± 0.47 **

Data are expressed as means ± SD (*n* = 10) ** *p* < 0.01 versus vehicle group. SOD, superoxide dismutase; GPX, glutathione peroxidase; MDA, malondialdehyde. WOPs-LG, walnut oligopeptides low-dose group; WOP-MG, walnut oligopeptides medium-dose group; WOP-HG, walnut oligopeptides high-dose group.

**Table 4:** Amino acid composition of WOPs.

Amino Acid	Amino Acid Composition of WOPs (g/100 g)
Asp	0.09
Glu	0.12
Ser	0.11
His	0.02
Gly	0.06
Thr	0.15
Arg	1.02
Ala	0.13
Tyr	0.30
Cys	0.01
Val	0.07
Met	0.02
Phe	0.48
Ile	0.04
Leu	0.28
Lys	0.05
Pro	0.06

WOPs, small molecule oligopeptides isolated from walnut.

## References

[1] Penner I.K., Paul F. (2017). Fatigue as a symptom or comorbidity of neurological diseases. Nat. Rev. Neurol..

[2] Mizuno K., Tanaka M., Nozaki S., Mizuma H., Ataka S., Tahara T., Sugino T., Shirai T., Kajimoto Y., Kuratsune H. (2008). Antifatigue effects of coenzyme Q10 during physical fatigue. Nutrition.

[3] Wei W., Li Z.P., Zhu T., Fung H.Y., Wong T.L., Wen X., Ma D.L., Leung C.H., Han Q.B. (2017). Anti-fatigue effects of the unique polysaccharide marker of *Dendrobium officinale* on BALB/c mice. Molecules.

[4] Duan F.F., Guo Y., Li J.W., Yuan K. (2017). Antifatigue effect of Luteolin-6-C-Neohesperidoside on oxidative stress injury induced by forced swimming of rats through modulation of Nrf2/ARE signaling pathways. Oxid. Med. Cell. Longev..

[5] Wan J.J., Qin Z., Wang P.Y., Sun Y., Liu X. (2017). Muscle fatigue: General understanding and treatment. Exp. Mol. Med..

[6] Jin G., Kataoka Y., Tanaka M., Mizuma H., Nozaki S., Tahara T., Mizuno K., Yamato M., Watanabe Y. (2009). Changes in plasma and tissue amino acid levels in an animal model of complex fatigue. Nutrition.

[7] Xu J., Potter M., Tomas C., Elson J.L., Morten K.J., Poulton J., Wang N., Jin H., Hou Z., Huang W.E. (2018). A new approach to find biomarkers in chronic fatigue syndrome/myalgic encephalomyelitis (CFS/ME) by single-cell Raman micro-spectroscopy. Analyst.

[8] Bao L., Cai X., Wang J., Zhang Y., Sun B., Li Y. (2016). Anti-fatigue effects of small molecule oligopeptides isolated from *Panax ginseng* C. A. Meyer in mice. Nutrients.

[9] Chen J., Wang X., Cai Y., Tang M., Dai Q., Hu X., Huang M., Sun F., Liu Y., Xia P. (2013). Bioactivity-guided fractionation of physical fatigue-attenuating components from *Rubus parvifolius* L.. Molecules.

[10] Li D., Ren J.W., Zhang T., Liu R., Wu L., Du Q., Li Y. (2018). Anti-fatigue effects of small-molecule oligopeptides isolated from *Panax quinquefolium* L. in mice. Food Funct..

[11] Chen Y.M., Lin C.L., Wei L., Hsu Y.J., Chen K.N., Huang C.C., Kao C.H. (2016). Sake protein supplementation affects exercise performance and biochemical profiles in power-exercise-trained mice. Nutrients.

[12] Zhao H.P., Zhang Y., Liu Z., Chen J.Y., Zhang S.Y., Yang X.D., Zhou H.L. (2017). Acute toxicity and anti-fatigue activity of polysaccharide-rich extract from corn silk. Biomed. Pharmacother..

[13] Chen N., Yang H., Sun Y., Niu J., Liu S. (2012). Purification and identification of antioxidant peptides from walnut (*Juglans regia* L.) protein hydrolysates. Peptides.

[14] Ma S., Huang D., Zhai M., Yang L., Peng S., Chen C., Feng X., Weng Q., Zhang B., Xu M. (2015). Isolation of a novel bio-peptide from walnut residual protein inducing apoptosis and autophagy on cancer cells. BMC Complement. Altern. Med..

[15] Poulose S.M., Bielinski D.F., Shukitt-Hale B. (2013). Walnut diet reduces accumulation of polyubiquitinated proteins and inflammation in the brain of aged rats. J. Nutr. Biochem..

[16] Liu M., Du M., Zhang Y., Xu W., Wang C., Wang K., Zhang L. (2013). Purification and identification of an ACE inhibitory peptide from walnut protein. J. Agric. Food Chem..

[17] Liao W., Zhang R., Dong C., Yu Z., Ren J. (2016). Novel walnut peptide-selenium hybrids with enhanced anticancer synergism: Facile synthesis and mechanistic investigation of anticancer activity. Int. J. Nanomed..

[18] Kim D.I., Kim K.S. (2013). Walnut extract exhibits anti-fatigue action via improvement of exercise tolerance in mice. Lab Anim. Res..

[19] He L.X., Wang J.B., Sun B., Zhao J., Li L., Xu T., Li H., Sun J.Q., Ren J.W., Liu R. (2017). Suppression of TNF-alpha and free radicals reduces systematic inflammatory and metabolic disorders: Radioprotective effects of ginseng oligopeptides on intestinal barrier function and antioxidant defense. J. Nutr. Biochem..

[20] Xu X., Ding Y., Yang Y., Gao Y., Sun Q., Liu J., Yang X., Wang J., Zhang J. (2018). β-glucan salecan improves exercise performance and displays anti-fatigue effects through regulating energy metabolism and oxidative stress in mice. Nutrients.

[21] Kan N.W., Ho C.S., Chiu Y.S., Huang W.C., Chen P.Y., Tung Y.T., Huang C.C. (2016). Effects of resveratrol supplementation and exercise training on exercise performance in middle-aged mice. Molecules.

[22] Zhao Y.Q., Zeng L., Yang Z.S., Huang F.F., Ding G.F., Wang B. (2016). Anti-fatigue effect by peptide fraction from protein hydrolysate of *Croceine croaker* (*Pseudosciaena crocea*) swim bladder through inhibiting the oxidative reactions including DNA damage. Mar. Drugs.

[23] Gobatto C.A., de Mello M.A., Sibuya C.Y., de Azevedo J.R., dos Santos L.A., Kokubun E. (2001). Maximal lactate steady state in rats submitted to swimming exercise. Comp. Biochem. Physiol. A Mol. Integr. Physiol..

[24] Lin Y., Liu H.L., Fang J., Yu C.H., Xiong Y.K., Yuan K. (2014). Anti-fatigue and vasoprotective effects of quercetin-3-*O*-gentiobiose on oxidative stress and vascular endothelial dysfunction induced by endurance swimming in rats. Food Chem. Toxicol..

[25] Chen Y.M., Wei L., Chiu Y.S., Hsu Y.J., Tsai T.Y., Wang M.F., Huang C.C. (2016). Lactobacillus plantarum TWK10 supplementation improves exercise performance and increases muscle mass in mice. Nutrients.

[26] Guo Z., Lin D., Guo J., Zhang Y., Zheng B. (2017). In vitro antioxidant activity and in vivo anti-fatigue effect of sea horse (hippocampus) peptides. Molecules.

[27] Powers S.K., Jackson M.J. (2008). Exercise-induced oxidative stress: Cellular mechanisms and impact on muscle force production. Physiol. Rev..

[28] Downs M.L., Semic-Jusufagic A., Simpson A., Bartra J., Fernandez-Rivas M., Rigby N.M., Taylor S.L., Baumert J.L., Mills E.N. (2014). Characterization of low molecular weight allergens from English walnut (*Juglans regia*). J. Agric. Food Chem..

[29] Allen D.G., Lamb G.D., Westerblad H. (2008). Skeletal muscle fatigue: Cellular mechanisms. Physiol. Rev..

[30] Paillard T. (2012). Effects of general and local fatigue on postural control: A review. Neurosci. Biobehav. Rev..

[31] Anand T., Phani Kumar G., Pandareesh M.D., Swamy M.S., Khanum F., Bawa A.S. (2012). Effect of bacoside extract from *Bacopa monniera* on physical fatigue induced by forced swimming. Phytother. Res..

[32] Wang X., Qu Y., Zhang Y., Li S., Sun Y., Chen Z., Teng L., Wang D. (2018). Antifatigue potential activity of *Sarcodon imbricatus* in acute excise-treated and chronic fatigue syndrome in mice via regulation of Nrf2-mediated oxidative stress. Oxid. Med. Cell. Longev..

[33] Zhao X.N., Liang J.L., Chen H.B., Liang Y.E., Guo H.Z., Su Z.R., Li Y.C., Zeng H.F., Zhang X.J. (2015). Anti-fatigue and antioxidant activity of the polysaccharides isolated from *Millettiae speciosae* Champ. Leguminosae. Nutrients.

[34] Robergs R.A., Ghiasvand F., Parker D. (2004). Biochemistry of exercise-induced metabolic acidosis. Am. J. Physiol. Regul. Integr. Comp. Physiol..

[35] Hsu Y.J., Huang W.C., Chiu C.C., Liu Y.L., Chiu W.C., Chiu C.H., Chiu Y.S., Huang C.C. (2016). Capsaicin supplementation reduces physical fatigue and improves exercise performance in mice. Nutrients.

[36] Ma G.D., Chiu C.H., Hsu Y.J., Hou C.W., Chen Y.M., Huang C.C. (2017). Changbai mountain Ginseng (*Panax ginseng* C.A. Mey) extract supplementation improves exercise performance and energy utilization and decreases fatigue-associated parameters in mice. Molecules.

[37] Wang S.Y., Huang W.C., Liu C.C., Wang M.F., Ho C.S., Huang W.P., Hou C.C., Chuang H.L., Huang C.C. (2012). Pumpkin (*Cucurbita moschata*) fruit extract improves physical fatigue and exercise performance in mice. Molecules.

[38] Armstrong C.W., McGregor N.R., Butt H.L., Gooley P.R. (2014). Metabolism in chronic fatigue syndrome. Adv. Clin. Chem..

[39] Mota M.R., Dantas R.A.E., Oliveira-Silva I., Sales M.M., Sotero R.D.C., Venancio P.E.M., Teixeira Junior J., Chaves S.N., de Lima F.D. (2017). Effect of self-paced active recovery and passive recovery on blood lactate removal following a 200 m freestyle swimming trial. J. Sports Med..

[40] Kent J.A., Ortenblad N., Hogan M.C., Poole D.C., Musch T.I. (2016). No muscle is an island: Integrative perspectives on muscle fatigue. Med. Sci. Sports Exerc..

[41] Osman W.N.W., Mohamed S. (2018). Standardized *Morinda citrifolia* L. and *Morinda elliptica* L. leaf extracts alleviated fatigue by improving glycogen storage and lipid/carbohydrate metabolism. Phytother. Res..

[42] Tan S.J., Li N., Zhou F., Dong Q.T., Zhang X.D., Chen B.C., Yu Z. (2014). Ginsenoside Rb1 improves energy metabolism in the skeletal muscle of an animal model of postoperative fatigue syndrome. J. Surg. Res..

[43] Manoharan P., Radzyukevich T.L., Hakim Javadi H., Stiner C.A., Landero Figueroa J.A., Lingrel J.B., Heiny J.A. (2015). Phospholemman is not required for the acute stimulation of Na(+)-K(+)-ATPase alpha(2)-activity during skeletal muscle fatigue. Am. J. Physiol. Cell Physiol..

[44] Wu R.M., Sun Y.Y., Zhou T.T., Zhu Z.Y., Zhuang J.J., Tang X., Chen J., Hu L.H., Shen X. (2014). Arctigenin enhances swimming endurance of sedentary rats partially by regulation of antioxidant pathways. Acta Pharmacol. Sin..

[45] Zamanian M., Hajizadeh M.R., Esmaeili Nadimi A., Shamsizadeh A., Allahtavakoli M. (2017). Antifatigue effects of troxerutin on exercise endurance capacity, oxidative stress and matrix metalloproteinase-9 levels in trained male rats. Fundam. Clin. Pharmacol..

[46] Ostojic S.M. (2016). Exercise-induced mitochondrial dysfunction: A myth or reality?. Clin. Sci..

[47] Milone M., Wong L.J. (2013). Diagnosis of mitochondrial myopathies. Mol. Genet. Metab..

[48] Vincent G., Lamon S., Gant N., Vincent P.J., MacDonald J.R., Markworth J.F., Edge J.A., Hickey A.J. (2015). Changes in mitochondrial function and mitochondria associated protein expression in response to 2-weeks of high intensity interval training. Front. Physiol..

[49] Meeus M., Nijs J., Hermans L., Goubert D., Calders P. (2013). The role of mitochondrial dysfunctions due to oxidative and nitrosative stress in the chronic pain or chronic fatigue syndromes and fibromyalgia patients: Peripheral and central mechanisms as therapeutic targets?. Expert Opin. Ther. Targets.

[50] Hsieh P.F., Liu S.F., Hung T.J., Hung C.Y., Liu G.Z., Chuang L.Y., Chen M.F., Wang J.L., Shi M.D., Hsu C.H. (2016). Elucidation of the therapeutic role of mitochondrial biogenesis transducers NRF-1 in the regulation of renal fibrosis. Exp. Cell Res..

[51] Chang C.K., Chang Chien K.M., Chang J.H., Huang M.H., Liang Y.C., Liu T.H. (2015). Branched-chain amino acids and arginine improve performance in two consecutive days of simulated handball games in male and female athletes: A randomized trial. PLoS ONE.

[52] Chen I.F., Wu H.J., Chen C.Y., Chou K.M., Chang C.K. (2016). Branched-chain amino acids, arginine, citrulline alleviate central fatigue after 3 simulated matches in taekwondo athletes: A randomized controlled trial. J. Int. Soc. Sports Nutr..

[53] Tan W., Yu K.Q., Liu Y.Y., Ouyang M.Z., Yan M.H., Luo R., Zhao X.S. (2012). Anti-fatigue activity of polysaccharides extract from Radix Rehmanniae Preparata. Int. J. Biol. Macromol..

